# Only transesophageal echocardiography guided patent foramen ovale closure: A single-center experience

**DOI:** 10.3389/fsurg.2022.977959

**Published:** 2022-10-11

**Authors:** Su Wang, Ge Zhu, Zhang Liu, Jian Zhou, Wangfu Zang

**Affiliations:** Department of Cardio-Thoracic Surgery, Shanghai Tenth People’s Hospital, School of Medicine, Tongji University, Shanghai, China

**Keywords:** patent foramen ovale, transesophageal echocardiography, closure, stroke, angiographic

## Abstract

**Background:**

An increasing number of studies have proved that patent foramen ovale (PFO) occlusion could reduce the incidence of recurrent stroke more than drug therapy alone under certain conditions. Which is the “best” guidance technique still remains to be discussed.

**Methods:**

A single center retrospective study enrolled 120 patients (mean age 52.51 ± 14.29 years) who underwent PFO closure between April 2019 and March 2021. 87 patients (72.5%) had suffered cryptogenic stroke (CS) at least one time, and 24 patients (20%) had repetitive episodes of hemicrania unsourced. 65 patients were in the transesophageal echocardiography (TEE) guidance group (T-group), and the other 55 patients were in the angiographic guidance group (A-group).

**Results:**

There were no significant differences in crucial clinical characteristics between the two groups. In T-group, the procedural success rate was higher (100% vs. 92.7%, *P* = 0.028), and the procedural time was shorter (23.15 ± 13.87 vs. 25.75 ± 7.19, *P* = 0.001). No difference was detected in the procedural complication rate. Follow-up were performed at least 12 months. At 12 months, new atrial fibrillation occurred in 1 patient (1.5%) in the T-group and in 1 patient (1.8%) in the A-group (*P* = 0.905). Residual shunt occurred in 1 patient (1.5%) in the T-group and in 3 patients (5.5%) in the A-group (*P* = 0.236). Recurrent cerebral ischemia occurred in 2 patient (3.1%) in the T-group and in 2 patients (3.6%) in the A-group (*P* = 0.865).

**Conclusion:**

The use of only intra-procedural TEE guidance for PFO closure is safe and effective. The whole procedure can be performed without fluoroscopy and contrast medium. The short and medium follow-up results are satisfactory, especially in the residual shunt.

## Introduction

Patent foramen ovale (PFO) occurs in about 25% of adults (1), most of whom do not require intervention. However, a growing number of studies have discovered a strong association between PFO and cryptogenic stroke (CS). CS is referred to a stroke whose causes cannot be found after complete clinical and instrumental evaluations, and it is a diagnosis of exclusion. The incidence of CS is approximately 25% of the total number of ischemic strokes (2), while the incidence of PFO is as high as 34%–77% in these populations (3, 4), which significantly higher than that in the general adult population.

Percutaneous transcatheter closure of the PFO as secondary prevention for CS was confirmed in many studies. The most common and minimally invasive procedure is percutaneous through the femoral vein with an angiographic guide (5). Fluoroscopy is required to guide the entire procedure.

The contrast medium was used to determine the position and shape of PFO when the guide could not pass through the defect. In addition to this, it was used to confirm the operation result after the occlude was implanted. However, x-rays can be harmful to the human body to some extent. Especially for certain special populations such as pregnant women and children, it may not be possible to perform the procedure in this way. In addition, the use of contrast medium puts some patients with renal impairment at risk for contrast-related kidney injury (6). Therefore, in order to reduce the discomfort of patients during surgeries and avoid the harm of contrast medium and fluoroscopy to special populations, it is may also a good choice to complete the procedures guided by transesophageal echocardiography (TEE) alone (7). It has been demonstrated that the use of TEE guidance alone is effective and safe for the interventional treatment of PFO (8).

The purpose of our study was to compare the outcomes of the two different guidance methods in PFO closure procedures and postoperative follow-up, which may provide a reference for doctors choosing methods.

## Materials and methods

120 patients matched the criteria undergoing percutaneous PFO closure between April 2019 and March 2021, 55 patients under angiographic guidance (A-group), and the others 65 patients under TEE guidance (T-group). Patients chose which group to go into mainly based on their willing after understanding the different guidance schemes between the two groups. Procedures in each group were performed by a single doctor who was similar in age, working years, and experience.

All patients underwent cerebral CT scan (or MRI) and cerebrovascular CTA. These examinations helped us confirm cardio-embolic characteristics. Besides it, a carotid and vertebral artery ultrasound Doppler examination was a requirement to exclude severe stenosis. An ECG was also a routine test. Patients with suspected arrhythmias especially atrial fibrillation should receive the 24 h Holter ECG at least one time.

Contrast transthoracic echocardiography (C-TTE) was performed on all patients. Blood activated normal saline was used as bubble contrast, and it was injected *via* the peripheral vein in pellet form (2–3 s). The severity of right-to-left shunt (RLS) was determined by observing the number of microbubbles in the left heart within 3–5 cardiac cycles after Valsalva maneuvers and the contrast medium entering the right heart (9). The protocol we used for shunt grading incorporated 4 grades: grade 1: 5 bubbles; grade 2: 5–25 bubbles; grade 3: 25 bubbles; and grade 4: opacification of chamber (10).

Patients enrolled in our study should meet al.l.the following criteria: (1) less than 70 years old; (2) cryptogenic stroke (or transient ischemic attack, TIA) at least one time or repetitive episodes of hemicrania unsourced; (3) no evidence of atrial fibrillation (AF); (4) the severity of RLS was grade 2–4. Exclusion criteria were infective diseases, pregnancy, and contraindication to antiplatelet or anticoagulant therapy.

All devices were the Amplatzer PFO Occluders (Abbott.). Two sizes of them were used: 18 and 25 mm. These were the most common sizes and could be used in most operations.

The closure procedure was performed with topical anesthesia in A-group. We delivered the occluder through the femoral vein by the method of percutaneous under fluoroscopy. the contrast medium was used to determine the position and shape of PFO. If the PFO could not be seen by simply contrast medium injected, patients were instructed to perform Valsalva maneuvers (11). Operation time, fluoroscopy time, and the dosage of contrast medium (iodixanol) were recorded. And the number of necessary physicians of A-group usually has one main surgeon and one assistant, plus a nurse and one in charge of machine adjustment.

**Figure 1:**
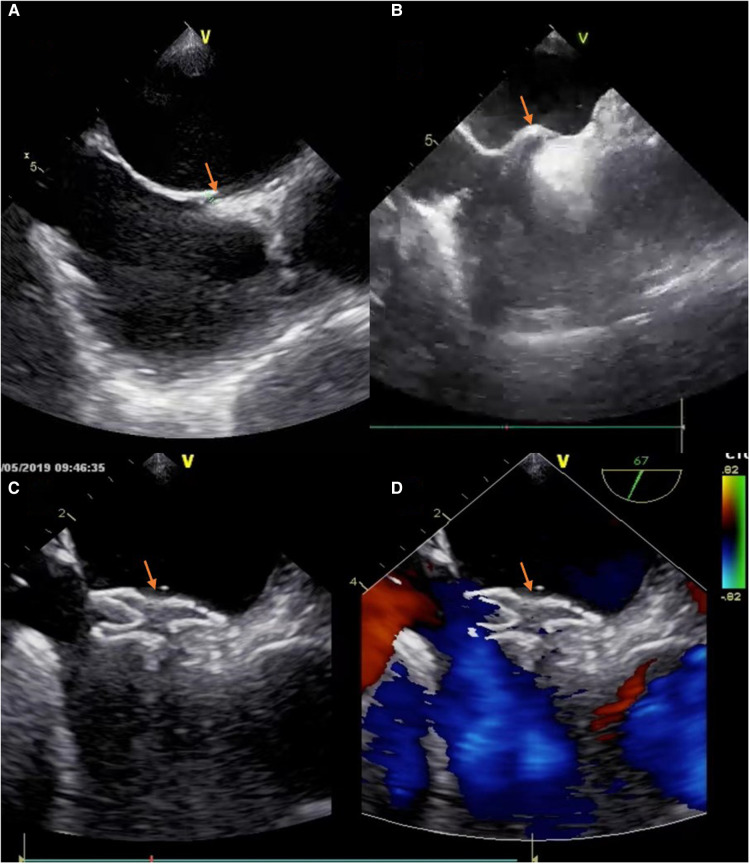
The process of deploying a PFO occluder. (**A**) The tunnel of PFO was appeared in TEE (“yellow arrow”). (**B**) The atrial septum was jacked up like a “tent” by a catheter (“yellow arrow”). (**C**) The device was positioned well (“yellow arrow”). (**D**) No residual colour flow crossed the atrial septum after the device was deployed (“yellow arrow”).

Patients needed to receive intratracheal intubation anesthesia in the T-group. We used the same operative pathway as A-group. The catheter reached the right atrium and guided into the PFO under real-time TEE, then the guidewire guided the catheter into the left atrium. After the occluder was released, TEE was used to evaluate the shape of the occluder and residual shunt (Figure 1). And the number of necessary physicians of T-group usually has one main surgeon and one assistant, plus a nurse and an anesthesiologist (also responsible for transesophageal echocardiography).

Before starting the procedures, heparin was injected (1 mg/kg) in order to keep an activated clotting time >250 s during the procedure. After the device implantation, dual antiplatelet therapy (aspirin 90 mg daily + clopidogrel 50 mg daily) was adopted for 6 months if there were no contraindications (such as severe bleeding) (12, 13). At 6 months, the continued antiplatelet therapy depended on the outcome of follow-up and discretion of the clinician. If patients need anticoagulant therapy during follow-up, such as new atrial fibrillation, a novel oral anticoagulant (NOAC) plus an antiplatelet medium were recommended.

All patients underwent follow-up for at least 6 months. Routine follow-up was performed at 1 and 6 months postoperatively. ECG and C-TTE were required items, and a cerebral CT scan was recommended if necessary. Patients with any recurrent neurologic symptoms would receive an evaluation by a neurological physician. The endpoints included death (cardiac and non-cardiac), recurrent cerebral ischemia, new atrial fibrillation, and residual shunt. Residual shunt is defined as RLS with grade 2–4.

Continuous variables were expressed as mean standard deviation or as median (interquartile range) and compared using the student's *t*-test or the Mann–Whitney test, as appropriate. The chi-square test or Fisher's exact test was performed for categorical variables. All the tests were two-tailed, and a value of *P* < 0.05 was considered statistically significant. All analyses were performed using SPSS 20.0 (SPSS, Chicago, IL, USA).

## Results

The baseline clinical characteristics of the two groups are shown in Table 1.

**Table 1:** Baseline clinical characteristics.

	T-group (*N* = 65)	A-group (*N* = 55)	*P*
Female (case/%)	24 (36.9)	27 (49.1)	/
Age (years)	53.38 ± 13.33	51.47 ± 15.396	0.602
Hypertension (cases/%)	29 (44.6)	25 (45.5)	0.927
Diabetes (cases/%)	17 (26.2)	15 (27.3)	0.891
Hyperlipidemia (cases/%)	8 (12.3)	5 (9.1)	0.574
CAD (cases/%)	4 (6.2)	5 (9.1)	0.544
TIA (cases/%)	8 (12.3)	6 (10.9)	0.813
Hemicrania (cases/%)	9 (13.8)	10 (18.2)	0.519
Single CS (cases/%)	37 (56.9)	26 (47.3)	0.294
Recurrent CS (cases/%)	12 (18.5)	12 (21.8)	0.648

Values are expressed as mean ± SD, median (interquartile range, 25%–75%) or *n* (%). CAD, coronary artery disease; TIA, transient ischemic attack; CS, cryptogenic stroke.

Between April 2019 and March 2021, 120 patients underwent PFO closure in our hospital. 55 patients were in A-group, and the other 65 patients were in T-group, according to their willingness after learning the two differences. Mean age was 52.51 ± 14.29 years, and 51 patients (42.5%) were female. 101 patients (84.2%) had suffered cryptogenic stroke (or TIA) at least one time, and 19 patients (15.8%) had repetitive episodes of hemicrania unsourced.

There were no significant differences in crucial clinical characteristics between the two groups.

The procedural characteristics of the two groups are reported in Table 2.

**Table 2:** Procedural characteristics.

	T-group (*N* = 65)	A-group (*N* = 55)	*P*
Procedure success (case/%)	65 (100)	51 (92.7)*	0.028
AF (case/%)	0 (0)	0 (0)	/
PE (case/%)	2 (3.1)	0 (0)	0.191
CI (case/%)	0 (0)	0 (0)	/
Operation time (minutes)	23.15 ± 13.87	25.75 ± 7.19**	0.001
Postoperative hospital stay (days)	2.28 ± 0.74	2.38 ± 0.56	0.131
Fluoroscopy time (minutes)	/	15.91 ± 6.52	/
Contrast medium (ml)	/	62.82 ± 34.96	/

Values are expressed as mean ± SD, median (interquartile range, 25%–75%) or *n* (%). AF, atrial fibrillation; PE, pericardial effusion; CI, cerebral ischemia.

* Compared with T-group, *P* < 0.05.

** Compared with T-group, *P* < 0.01.

In A-group, 4 patients were terminated because their PFOs could not be found or passed through during procedures. The operation success rate was lower in this group. The operation time of the A-group was longer (25.75 ± 7.19 A-group vs. 23.15 ± 13.87 T-group, *P* = 0.001), while the total operation time (including anesthesia time) of the T-group was longer. There was no perioperative atrial fibrillation and cerebral ischemia in both groups. pericardial effusion was observed in 2 patients (3.1%) in T-group, one of which resulted in thoracotomy. The postoperative hospital stay time was no difference between 2 groups. And obviously, the fluoroscopy and contrast medium only existed in A-group, the fluoroscopy time was (15.91 ± 6.52) mins and the contrast medium was (62.82 ± 34.96) ml.

The short-term and middle-term follow-up data have resulted in Table 3.

**Table 3:** Short-term and middle-term follow-up.

	T-group (*N* = 65)	A-group (*N* = 55)	*P*
RCI 1 month (case/%)	0 (0)	0 (0)	/
RCI 6 months (case/%)	1 (1.5)	1 (1.8)	0.905
RCI 12 months (case/%)	2 (3.1)	2 (3.6)	0.865
AF 1 month (case/%)	1 (1.5)	0 (0)	0.358
AF 6 months (case/%)	1 (1.5)	1 (1.8)	0.905
AF 12 months (case/%)	1 (1.5)	1 (1.5)	0.905
RS 1 month (case/%)	2 (3.1)	5 (9.1)	0.163
RS 6 months (case/%)	1 (1.5)	4 (7.3)	0.119
RS 12 months (case/%)	1 (1.5)	3 (5.5)	0.236
Cardiac death 12 months (case/%)	0 (0)	0 (0)	/
Non-cardiac death 12 months (case/%)	0 (0)	0 (0)	/

Values are expressed as mean ± SD, median (interquartile range, 25%–75%) or *n* (%). RCI, recurrent cerebral ischemia; AF, atrial fibrillation; RS, residual shunt.

Follow-up were performed at least 12 months. Routine follow-up was carried out at 1, 3 and 12 months postoperatively. At 1 month, new atrial fibrillation came out in T-group (1.5%), and there was no new cerebral ischemia or death. Residual shunt occurred higher in A-group, although there was no significant difference between them (9.1% A-group vs. 3.1% T-group, *P* = 0.163). At 6 months, new atrial fibrillation came out in A-group (1.8%). Residual shunt (5.5% A-group vs. 1.5% T-group, *P* = 0.236) and recurrent cerebral ischemia (1.8% A-group vs. 1.5% T-group, *P* = 0.905) occurred in both groups, but there was no significant difference between them. At 12 months, new atrial fibrillation occurred in 1 patient (1.5%) in the T-group and in 1 patient (1.8%) in the A-group (*P* = 0.905). Residual shunt occurred in 1 patient (1.5%) in the T-group and in 3 patients (5.5%) in the A-group (*P* = 0.236). Recurrent cerebral ischemia occurred in 2 patient (3.1%) in the T-group and in 2 patients (3.6%) in the A-group (*P* = 0.865).

## Discussion

The CLOSE (14), REDUCE (15) and RESPECT (16) studies about the closure of PFO with occluders were published on the New England Journal of Medicine journal in 2017, which proved that PFO occlusion could reduce the incidence of recurrent stroke more than drug therapy alone on certain conditions. Since then, many authoritative studies have been published, and guidelines in Europe (12), the United States (17), and even Asia Pacific (18) have been updated to support the use of PFO closure in the appropriate population. Therefore, our study is no longer focusing on the meaning and appropriate people of PFO closure, but discussing the safety and feasibility of a new guidance method with no angiographic guidance.

The main findings of this study are as follows: (1) the PFO closure *via* a femoral vein under intra-procedural TEE alone guidance is safe and effective, and the short and medium follow-up results are satisfactory; (2) patients who are unable to tolerate contrast medium (allergies or renal failure) can also have chance to undergo PFO closure surgery; (3) TEE guidance alone in surgery is an option for patients who are concerned about the damage from fluoroscopy.

The angiographic-only guidance is a “classic” technique. This method makes it possible to finish the operation by local anesthesia. The overall operative path is relatively straightforward, so it is considered an ideal guidance approach. However, fluoroscopy alone will not allow the visualization of intraoperative intracardiac thrombus, a rare but important procedural complication (19). Moreover, this approach will be more difficult for PFO patients with small shunt or long tunnel (≥8 mm) or multifenestrated interatrial septum (IAS) (20), and may even lead to the failure of the operation. Continuous monitoring (e.g., TEE) is more appropriate for this kind of patient. Therefore, in order to reduce radiological exposure, some centers prefer the use of echocardiographic guidance as a supplement with the angiographic guidance. While others believe TEE in surgery may consume time and let it difficult to reduce the radiological exposure for both patients and surgeons, even providing useless or confounded information for surgeons making decisions (21).

The approach is TEE-only guidance without any other assistance in this study. And apparently, this method completely avoids the use of fluoroscopy and contrast medium. Most importantly, it improved the success rate of operation. This is mainly due to the special advantages of TEE: more accurate and real-time to recover the image of the atrial septum and analyze the shape and location of PFO. Because the shunt of PFO is very small in many cases, the contrast medium through RLS cannot be clearly identified during angiography, and even when Valsalva maneuver was released. The amount of contrast medium entering the left atrium through the PFO is very small, and the PFO path cannot be adequately detected. While in this case, our method can still assist in finishing the operation.

In our study, the mean total operation time was longer in T-group, because of general anesthesia. If the anesthesia time was not calculated, the procedural time was significantly shorter in T-group than in A-group. We used general anesthesia with tracheal intubation, in which 1% propofol 2 mg/kg, sufentanil 0.3 µg/kg, and rocuronium 0.6 mg/kg were used during induction of anesthesia. And 2% sevoflurane was used for anesthetic maintenance. At the end of surgery, 2 mg Neostigmine and 1 mg atropine were given to antagonize the residual effect of muscarinic drugs after the patient's spontaneous breathing resumed. This type of anesthesia is commonly used in general anesthesia for cardiovascular diseases, which has a low impact on the patient's blood pressure and heart rate and has the characteristics of rapid anesthesia and quick recovery (22). Also, no anesthesia-related complications were found in this study. General anesthesia (with or without endotracheal intubation) was used to avoid the discomfort and tension of patients and improve operation safety. The discomfort and tension came from three sources: (1) the pain from puncture (the inner size of sheath was 9F); (2) stimulation from TEE; (3) anxiety during operation. All the feelings will increase with the operation time extension.

In this study, pericardial effusion was observed in two patients in T-group, but we found no difference between the 2 groups regarding the PE(*P* = 0.191). The first of which occurred in the initial period of this study and eventually result in thoracotomy. During pericardium exploration, we found that the bleeding came from the left atrial appendage. The device was implanted deeply and released into the left atrial appendage, resulting in atrial appendage damage and pericardium tamponade. Due to the instant treatment, the patient was discharged without sequelae. The second patient did not undergo thoracotomy because of self-limiting bleeding. After the operation, 300 ml blood was drained through pericardiocentesis. Sauza mentioned the causes of pericardial effusion in TEE guidance: the main reason was that TEE did not recognize the top of the guidewire and transmission catheter in time (23). The atrial wall is thin and can be easily damaged, leading to bleeding. After we paid attention to this problem, we started to make sure that every step was carried out under the real-time TEE guidance, keep eyes on the top of the guidewire and transmission catheter, and the pericardial effusion didn't happen again. Using this approach is generally considered to place higher requirements on both the operator and the sonographer. The operator should have a good understanding of the heart structure under the ultrasound image, and the sonographer should be able to switch sections quickly to track the guidewire and catheter cooperated with the operator.

The other benefit of TEE guidance is related to a significant reduction in adverse events in follow-up, especially in reintervention (10.6% vs. 1.4%, *P* = 0.001) (24). This is mainly because of a lower incidence rate of residual shunt (16.8% vs. 8.0%, *P* = 0.015). In our study the A-group experienced a higher rate of significant residual shunt in 1 month (9.1% vs. 3.1%, *P* = 0.163), 6 months (7.3% vs. 1.5%, *P* = 0.119), 12 months (5.5% vs. 1.5%, *P* = 0.236). According to the statistics expert, the lack of statistical difference may be due to the limitation of sample size. During the operation, TEE helps measure defect size, ensure device fitting, and assess the resident shunt immediately (24). In previous studies, most patients enrolled were anatomically simple in the atrial septum (25), which supported the use of radiography alone to finish operations. However, some complex atrial septal structures are difficult to detect by angiographic guidance alone. And it will result in a higher rate of residual shunt.

## Limitations

The main limitation of this study is that it is not a randomized controlled trial. Fortunately, there was no statistically significant difference in the baseline clinical characteristics of the two groups, although it was up to the patients themselves to decide which group to enroll in. In addition, another limitation is that the sample size is limited and the follow-up time is not long enough, so the evidence needs to be further strengthened.

## Conclusion

The use of only intra-procedural TEE guidance in PFO closure is safe and effective. The method avoids fluoroscopic exposure and contrast medium, as well as presents a lower rate of residual shunt. It was hoped that the rate of reintervention would descend with this guidance.

## Data Availability

The raw data supporting the conclusions of this article will be made available by the authors, without undue reservation.
